# Supplementary material for: Effects of Bifidobacterium longum and Lactobacillus rhamnosus on Gut Microbiota in Patients with Lactose Intolerance and Persisting Functional Gastrointestinal Symptoms: A Randomised, Double-Blind, Cross-Over Study
Source: Nutrients. 2019 Apr 19;11(4):886. doi: 10.3390/nu11040886 (PMC6520754; doi:10.3390/nu11040886)
Supplement: Supplementary file 1 [file nutrients-11-00886-s001.pdf]

Materials and Methods

Culturable microbiota

Fecal samples (5 g) were mixed with 45 ml sterilized physiological solution and homogenized. Counts of viable bacterial cells were carried out as described by De Angelis et al. [1]. The following selective media were used: Wilkins-Chalgren anaerobe agar (total anaerobes); Plate count agar (total aerobes and anaerobes); MRS agar (*Enterococcus, Lactobacillus* and *Leuconostoc*); Slanetz and Bartley (*Enterococcus*); Rogosa agar, plus 1.32 ml/l of glacial acetic acid (*Lactobacillus*); M17 (*Lactococcus* and *Streptococcus*); Baird Parker (Staphylococcus); Wilkins-Chalgren anaerobe agar, plus GN selective supplements and sheep blood defibrinated (*Bacteroides, Porphyromonas* and *Prevotella*); MacConkey agar No2 (Enterobacteriaceae); GSP agar (Sigma-Aldrich, St. Louis, MO, USA), plus penicillin-G (60 g/l) (Pseudomonas, Aeromonas); Bifidobacterium agar modified (*Bifidobacterium*) (Becton Dickinson, Le Pont de Claix, SA, France). Except for *Bifidobacterium* agar modified, Chromocult and GSP agar, all media were purchased by Oxoid Ltd (Basingstoke, Hampshire, England).

Intestinal permeability

Intestinal permeability was assessed by oral administration of four sugar probes, which selectively characterize the permeability from different tracts of the gastrointestinal system [2]. Sucrose (SO) was used as a marker of gastro-duodenal permeability; lactulose (LA) and mannitol (MA) as LA/MA ratio were used as markers of small intestinal permeability, and sucralose (SU) as marker of colonic permeability. Subjects avoided carbohydrate-enriched diets and alcohol intake during the 24-h prior and during the 6-hr test period. In addition, the administration of anti-secretory drugs, probiotics, prebiotics, anti-inflammatory drugs and aspirin (if feasible) one week before the test, to avoid any interference with the gastrointestinal function. After 8-h overnight fasting, a pre-test urine sample was collected early in the morning. Subsequently, a water solution containing sucrose (20 g), mannitol (1 g), lactulose (5 g), and sucralose (1 g) mixed in 200 mL of water was administrated to each subject and drunk in 5 min. Urine sample was collected thereafter for the following 6 hours in a standard 2,000 mL plastic urine container. No food was allowed during the test, although water was allowed 1 hour after sugar ingestion. At the end of the 6 hours period the volume of the urine collection was recorded and sugar concentrations were measured by triple quadrupole mass spectrometry (Waters TQD) interfaced with HPLC (Waters Acquity UPLC). The fraction of the excreted sugars was the amount of sugar given to each subject and expressed as a percentage value. Values of sugars recovery above the following cut-off values were considered as abnormal results: sucrose recovery > 0.15%; LA/MA ratio > 0.03; sucralose recovery > 1.50%.

Results

Probiotics and vitamin B6 affects the fecal microbiota of LI patients

Table S3 shows the main microbial groups in faecal samples at T0 and after 30 days with treatment (ZR) and placebo (PL). No statistical difference existed between ZR and PL according to total anaerobes, *Lactococcus* and *Streptococcus*, *Staphylococcus*, *Bacteroides*, *Porphyromonas* and *Prevotella*, *Enterobacteria*, *Aeromonas* and *Pseudomonas*. Compared to T0, the treatment ZR increased the heterotrophic aerobic and anaerobic bacteria, presumptive lactic acid bacteria and Bifidobacterium.

References

1. De Angelis, M.; Piccolo, M.; Vannini, L.; Siragusa, S.; De Giacomo, A.; Serrazzanetti, D.I.; Cristofori, F.; Guerzoni, M.E.; Gobbetti, M.; Francavilla, R. Fecal microbiota and metabolome of children with autism and pervasive developmental disorder not otherwise specified. PLoS One **2013**; 8, e76993.
2. Del Valle-Pinero, A.Y.; Van Deventer, H.E.; Fourie, N.H.; Martino, A.C.; Patel, N.S.; Remaley, A.T.; Henderson, W.A. Gastrointestinal permeability in patients with irritable

bowel syndrome assessed using a four probe permeability solution. Clinica Chimica Acta **2013**; 418, 97-101.

Table S1. Amounts of daily micro/macronutrients at baseline

Nutrients	Intake
Carbohydrates (%)	41.64±1.29
Protein, total (%)	18.49±0.71
Animal protein (%)	54.5±2.9
Dairy protein (%)	8.1±0.83
Plant protein (%)	37.3±2.9
Lipids (%)	30.7±0.88
Dairy lipid (%)	7.15±0.83
Saturated fatty acids (%)	34.9±1.18
Fiber (g)	9.1±0.73
Olive oil (g)	23±2.79
Vitamin B6 (mg)	0.84 ± 0.06

Data are mean ± SE

Table S2. Changes of gastrointestinal symptoms and Bowel habits in Placebo and Treatment (ZR) according to sequence (1 = Placebo/ZR and 2 = ZR/Placebo) and globally

Parameter	Treatment	Sequence 1<br>(PL-ZR) N= 12	Sequence 2<br>(ZR-PL) N=11	Global<br>N=23	P-value
Bloating	Placebo	77 ± 5	82 ±3	77 ± 4	
	Treatment	62 ± 5	59 ± 6	60 ± 5	
	Placebo-treatment	-9 ± 5	-24±6	-17 ± 5	P=0.028
Abdominal pain	Placebo	56 ± 7	48 ± 7	53 ± 7	
	Treatment	40 ± 5	38 ± 7	39± 6	
	Placebo-treatment	-15 ± 5	-10 ± 11	-13±10	NS
Bristol score	Placebo	3 ± 0	3 ± 0	3± 0	
	Treatment	4 ± 0	4 ± 0	4 ± 0	
	Placebo-treatment	1 ± 0	1± 0	0 ± 0	NS

Data are mean ± SE

Table S3. Median values and range of cultivable of bacterial cells (log CFU/g) of the main microbial groups in the faecal samples of lactose intolerant patients at baseline (T0), after 30 days of treatment (ZR) or Placebo (PL).

**Cultivable bacteria**	**T0**	**PL**	**ZR**	**P value T0vsPL**	**P value T0vsZR**	**P value PLvsZR**
Heterotrophic aerobic and anaerobic bacteria	8.38	8.37	9.20	0.496	0.036	0.005
Total aenarobes	5.28	5.69	7.33	0.273	0.093	0.307
Lactic acid bacteria	6.54	6.49	8.32	0.431	0.011	0.012
Lactococcus and *Streptococcus*	7.70	7.44	8.01	0.184	0.288	0.167
Staphylococcus	5.68	5.33	6.66	0.316	0.067	0.084
Bacterioides, Porphyromonas and *Prevotella*	4.30	4.39	5.11	0.452	0.147	0.307
Enterobacteria	5.64	5.93	6.31	0.118	0.151	0.118
Aeromonas and Pseudomonas	4.71	5.05	5.86	0.199	0.384	0.378
Bifidobacterium	3.64	3.66	5.62	0.489	0.003	0.049
Enterococci	4.51	4.52	5.92	0.260	0.238	0.075

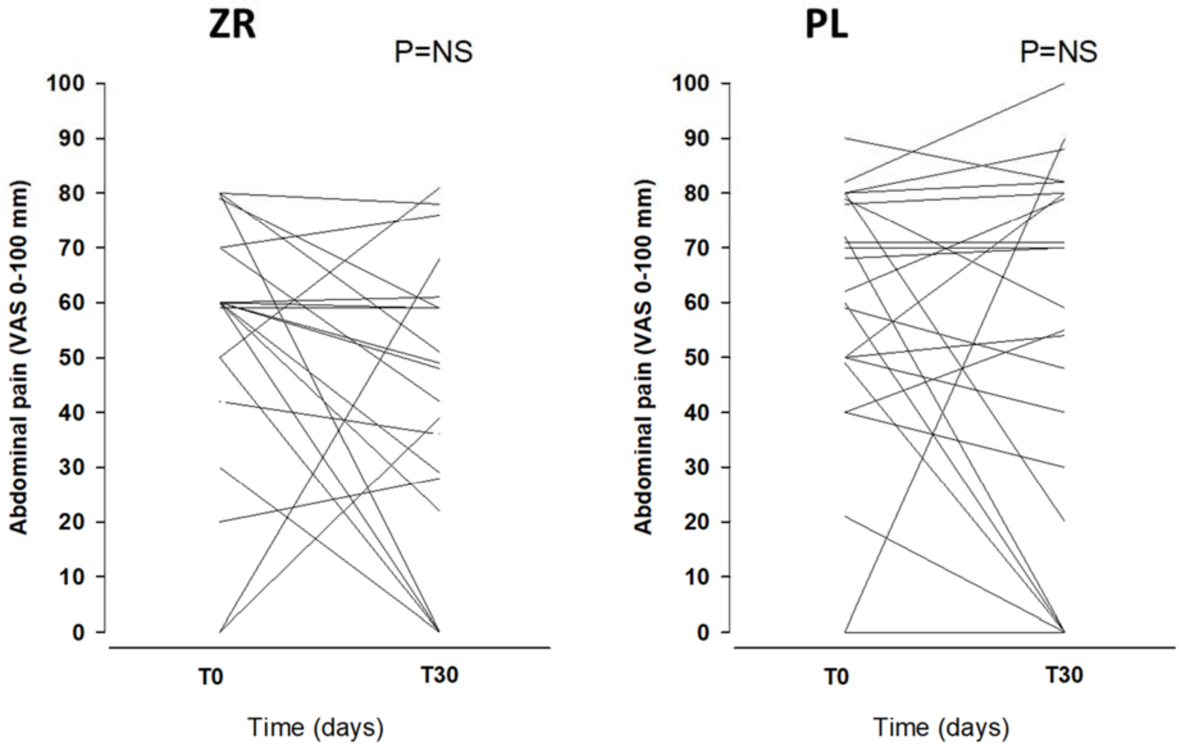

Figure S1. Representation of abdominal pain (VAS 0- 100 nm) in 23 patients at baseline (T0) and after 30 days (T30) of treatment (ZR) and Placebo (PL) by spaghetti graph.

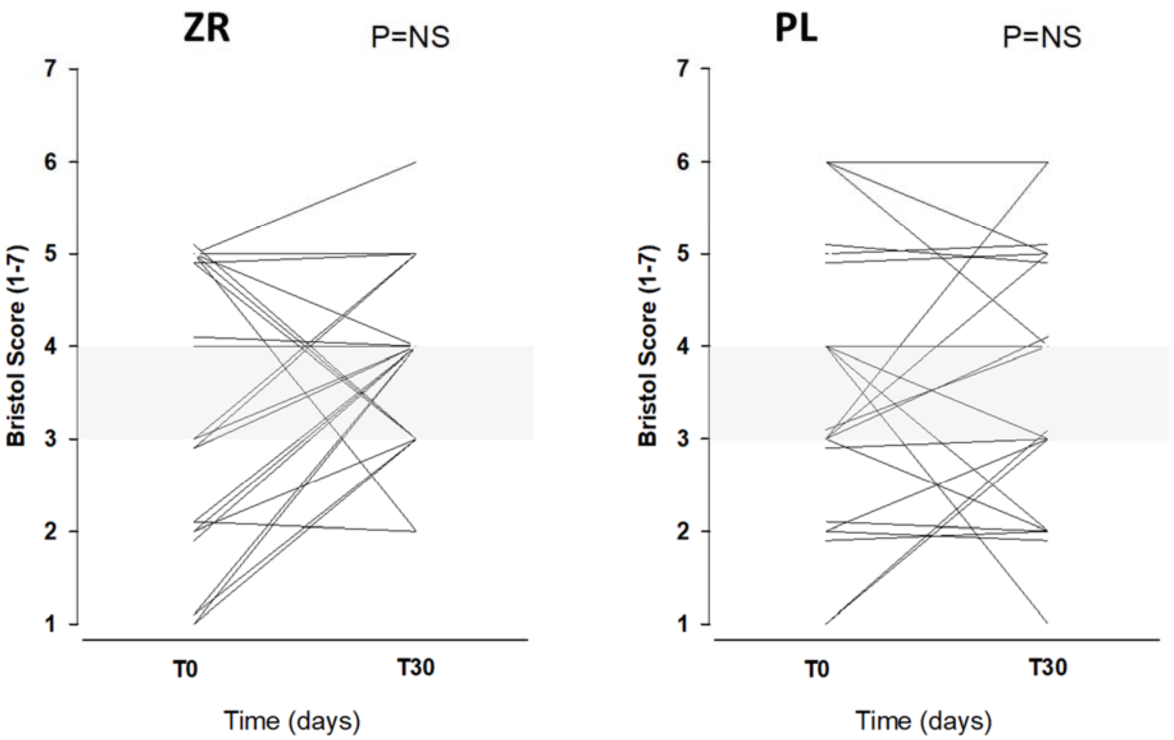

Figure S2. Representation of Bristol Score (1-7) at baseline (T0) in 23 patients and after 30 days (T30) of treatment (ZR) and Placebo (PL) by spaghetti graph.
